# Utility of F-18 FDG PET/CT for Detection of Bone Marrow Metastases in Prostate Cancer Patients Treated with Radium-223

**DOI:** 10.22038/aojnmb.2017.9896

**Published:** 2018

**Authors:** Kaoru Maruyama, Keita Utsunomia, Takahiro Nakamoto, Shigenari Kawakita, Takashi Murota, Noboru Tanigawa

**Affiliations:** 1 Departments of Radiology, Kansai Medical University, Japan; 2 Departments of Urology, Kansai Medical University, Japan

**Keywords:** Bone marrow, PET, Prostate cancer, Radiotherapy, Radium

## Abstract

A 76-year-old man with symptomatic bone metastases from castration-resistant prostate cancer underwent Radium-223-dichloride (Ra-223) therapy. Before Ra-223 therapy, he had normal peripheral blood cell counts. Ra-223 therapy relieved his shoulder and low back pain. The elevation of the serum prostate-specific antigen (PSA), doubling every month during Ra-223 therapy, suggested a PSA flare or relapse. Some lesions showed decrease and some lesions showed increase on Tc-99m hydroxymethylene diphosphonate bone scintigraphy at two weeks after the third injection of Ra-223 therapy. Ra-223 therapy was discontinued due to thrombocytopenia that was getting worse rapidly. After treatment discontinuation, namely four weeks after the third injection of Ra-223, F-18 fluorodeoxyglucose (FDG) Positron Emission Tomography (PET)/CT and a biopsy were performed to evaluate for metastases, and bone marrow metastases were found. Ra-223 was effective for osteoblastic lesions, but not for bone marrow metastases. FDG PET/CT, but not a Tc-99m based bone scan, detected diffuse bone marrow involvement by cancer. This case report is the first to clarify the utility of FDG PET for the detection of bone marrow metastases confirmed by pathological examination in Ra-223 therapy for progressive castration-resistant prostate cancer.

## Introduction

Radium-223-dichloride (Ra-223) is a radiotherapeutic agent for the treatment of painful osseous lesions from metastatic castration-resistant prostate cancer without visceral metastatic disease. Ra-223, similar in metabolism to Tc-99m based bone scan agents (1), mimics calcium and forms complexes with hydroxyapatite at areas of increased bone turnover, such as bone metastases. Ra-223 emits primarily alpha particles that have a short range (<100 μm) and affect osteoblastic lesions in bone metastases and may contribute to improving survival, palliating bone pain, and low bone marrow toxicity (2, 3). A case in which F-18 fluorodeoxyglucose (FDG) PET/CT had an impact on clinical management when a discrepancy occurred between the serum prostate-specific antigen (PSA) and the Tc-99m hydroxymethylene diphosphonate (HMDP) bone imaging response with relief of pain is reported. The potential utility of FDG PET/CT for detection of occult metastases in patients with PSA relapse during Ra-223 therapy and negative results on standard imaging studies (Tc-99m based bone scintigraphy) is suggested.

## Case report

**Figure 1:**
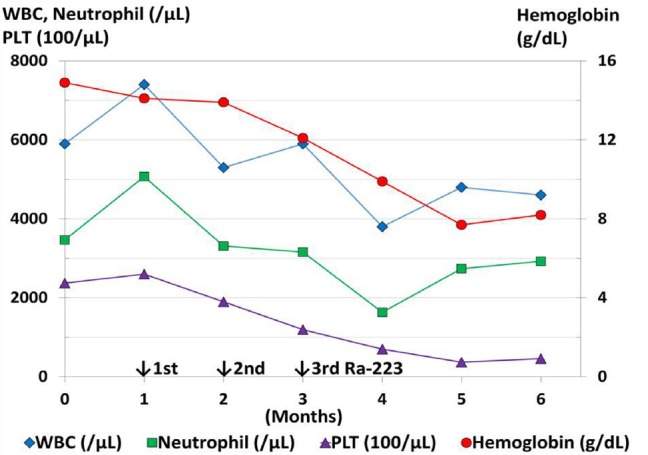
Peripheral blood cell counts before, during, and after Ra-223 treatment. White and red blood cell (WBC, RBC, respectively) and platelet (PLT) counts are normal before and decreased during and after Ra-223 therapy. The patient has thrombocytopenia (with PLT of 11.9×10^4^/μL at the third injection of Ra-223 therapy) and anemia (with a hemoglobin of 9.9 g/dL four weeks after the third injection of Ra-223 therapy), which rapidly gets worse. He became transfusion-dependent, presenting with a hemoglobin of 7.7 g/dL and requiring monthly red-cell transfusions.

A 76-year-old man with symptomatic bone metastases from hormone-refractory prostate cancer underwent Ra-223 therapy (3 of the planned 6 intravenous injections at a dose of 55 kBq per kg body weight, every 4 weeks). Before Ra-223 therapy, his hemoglobin was 14.9 g/dL, with a leukocyte count of 5900/μL and a platelet count of 24×10^4^/μL; all were normal (Figure 1). The serum testosterone level was 0.26 ng/ml. Abdominal pelvic CT was performed to document the presence of unequivocal visceral metastases before Ra-223 therapy.

**Figure 2:**
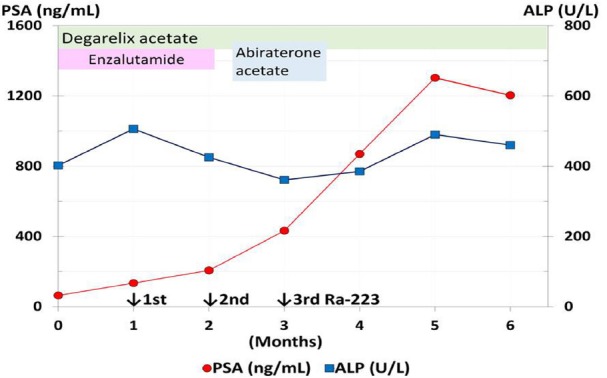
Prostate-specific antigen (PSA) and serum total alkaline phosphatase (ALP, marker indicating osteoblastic activity) trends and the response to Ra-223, with concomitant use of hormonal therapy. The PSA level is 64 ng/ml before Ra-223 therapy, and it increases by twice a month, with PSA peaking at 1303 ng/ml eight weeks after the third injection of Ra-223 therapy. ALP decreases from 506 to 361 U/L during Ra-223 therapy, compatible with a favorable response to radiotherapy and concordant with the general decrease of Tc-99m HMDP uptake in bone lesions.

**Figure 3:**
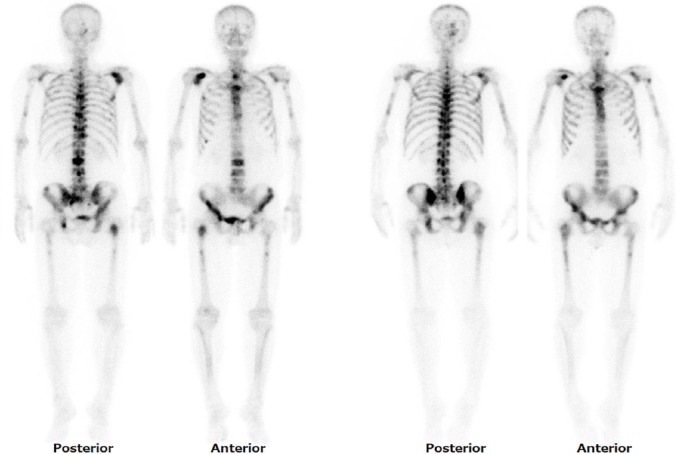
Tc-99m HMDP bone scintigraphy: images from left to right, pretreatment (posterior and anterior views) and 2 weeks after the third injection (posterior and anterior views) of Ra-223 therapy. Ra-223 therapy has somewhat reduced abnormal uptake on right shoulder and lumbar vertebra with pain relief, and somewhat increased uptake on some parts of skull, left jaw, sternum, thoracic vertebra, and patchy and mixed of increased and decreased uptake on humeri and femur in bone metastases. Meanwhile, the serum PSA level increases from 64 ng/mL before treatment to 869 ng/mL at 4 weeks after the third injection of Ra-223 therapy.

**Figure 4:**
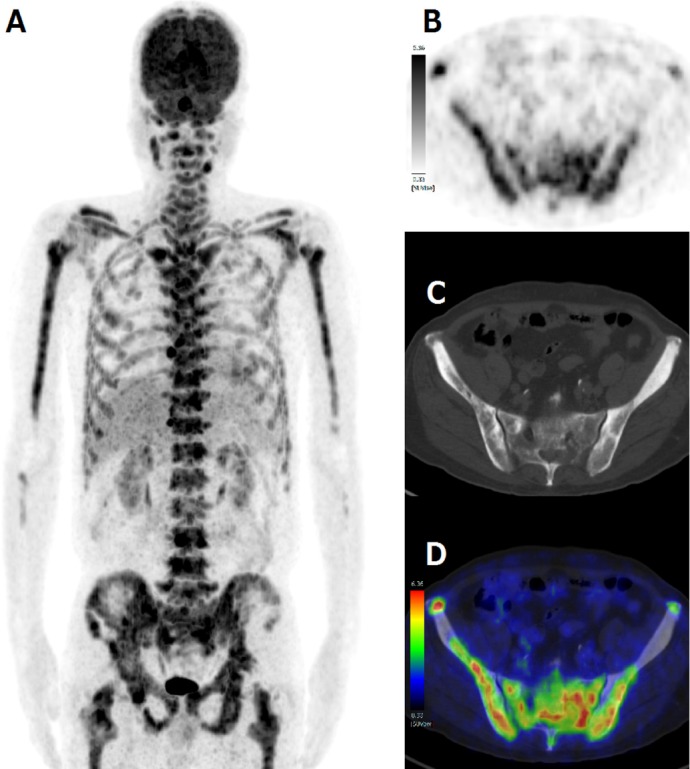
F-18 FDG PET maximum intensity projection (MIP) image (A), axial pelvic PET (B), CT (C), and fused PET/CT (D) at 4 weeks after the third injection of Ra-223 therapy. Diffuse FDG uptake in the bone without osteoblastic lesions shows active osteolytic metastases or bone marrow involvement. Osteoblastic lesions without FDG correspond to lesions with decreased HMDP uptake.

**Figure 5:**
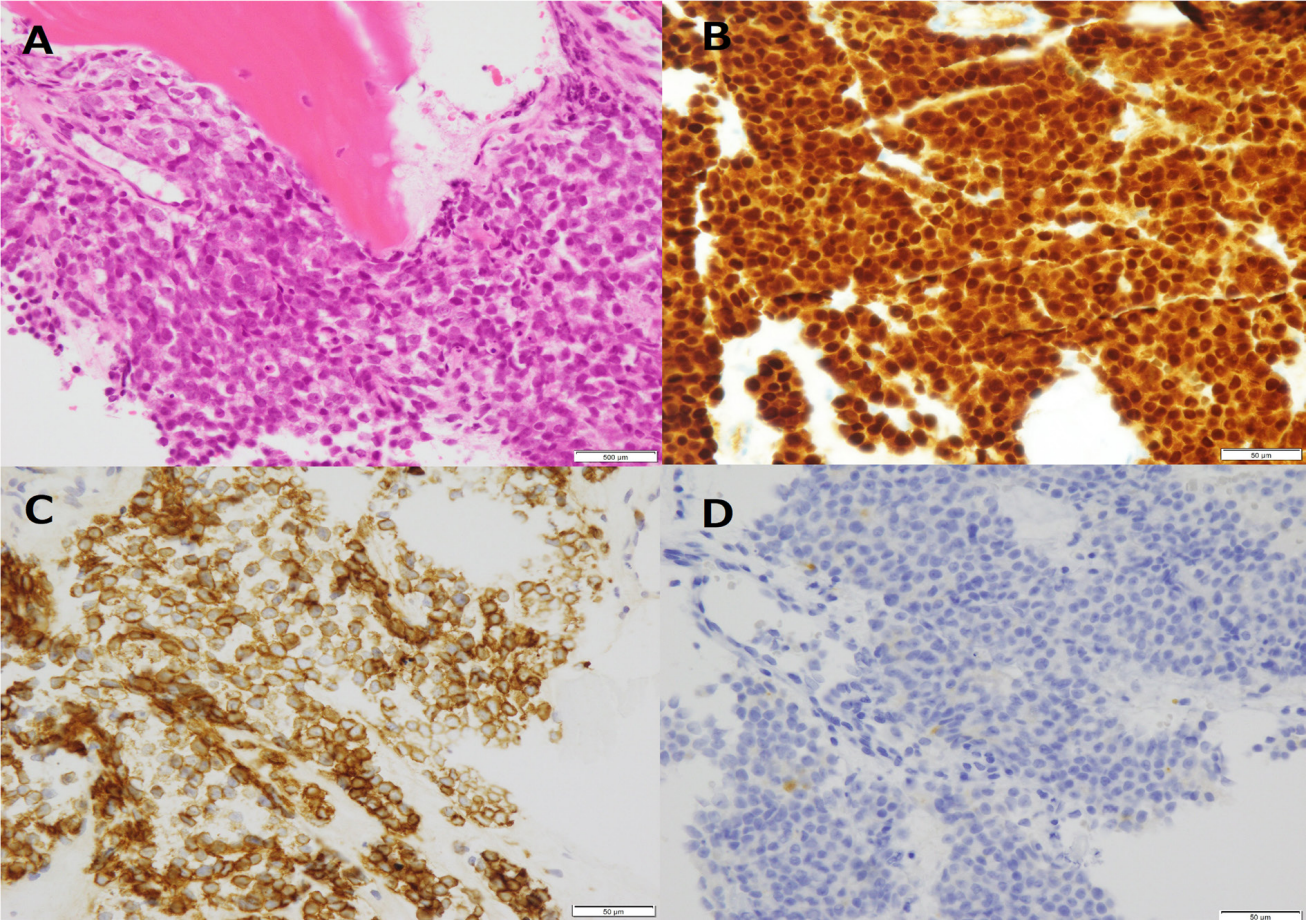
The images of a bone marrow biopsy are shown. Hematoxylin and eosin stain (A), ×400. Histologically, small round cells are diffusely spread in bone marrow. There is no ordinary tubular formation, as shown in adenocarcinoma of the prostate. On immunostaining, ×400, androgen receptor (B) and cytokeratin AE1/3 (C) are both positive. Strong androgen receptor nuclear staining (B) indicates histological malignancy. Positive immunostaining for prostate-specific antigen is confirmed in some cells (D), which is not inconsistent with low-grade prostatic carcinoma. The microscopic examination shows bone marrow infiltration with prostate carcinoma.

He had remained on androgen deprivation therapy with a novel endocrine agent, first enzalutamide and then abiraterone, with Ra-223 therapy (Figure 2). A decreased serum total alkaline phosphatase (ALP), PSA elevation (Figure 2) and blood cell count decreases (Figure 1) continued during Ra-223 therapy. PSA elevation suggested a PSA flare (4, 5) or relapse. Ra-223 therapy was discontinued due to thrombocytopenia (Figure 1). After the third injection of Ra-223, images showed that the decreased uptake in some parts corresponding to the pain relief lesion, and the increased uptake in some parts without pain on Tc-99m HMDP bone scintigraphy (Figure 3). To evaluate for metastases, FDG PET/CT was performed four weeks after the third injection of Ra-223 therapy and showed diffuse FDG uptake in bone without osteoblastic lesions (Figure 4). Bone marrow metastasis was confirmed by pathological examination following FDG PET/CT-guided bone marrow biopsy (Figure 5). He had received neither chemotherapy nor granulocyte-colony stimulating factor therapy that could have caused diffuse bone marrow FDG uptake.

## Discussion

This case report is the first to clarify the utility of FDG PET for the detection of bone marrow metastases confirmed by pathological examination in Ra-223 therapy for progressive castration-resistant prostate cancer. Quantitative analysis of bone scan and FDG PET could be used to make a management and therapeutic plan. Clinical implication of the utility of FDG PET/CT is as follows: FDG PET/CT for monitoring therapy response, for detection of relapse and for evaluation of disease activity, could be beneficial for prostate cancer patients on Ra-223 therapy. Before Ra-223 therapy, we checked no visceral metastases by only CT. We did not performed but recommend FDG PET/CT before Ra-223 therapy. There is no previous fully described research regarding FDG PET/CT in prostate cancer patients on Ra-223 therapy. Coleman et al. reported the effects of Ra-223 (intravenous injection every 4 weeks for 4 cycles) assessed by serial FDG PET imaging in advanced breast cancer patients (6). Response assessed with FDG PET/CT showed the benefit of the Ra-223 therapy for bone-metastatic breast cancer patients. Hsu et al. reported that FDG PET scans were useful tools for characterizing osteolytic and osteoblastic lesions in a prostate cancer mouse model (7). The role of FDG PET has been limited in the primary diagnosis or staging of prostate cancer due to low metabolic glucose metabolism or existence in the urinary tract. The usefulness of FDG PET in human prostate adenocarcinoma treated with Ra-223 therapy has been unclear because little information has been reported about the activity of newer drugs. This case highlights the impact of FDG PET on the detection and management of prostate cancer.

PSA is a marker of prostate cancer and is positive in bone marrow metastases (8). Burgio et al. reported that the PSA flare lasted a median of 28 days with abiraterone, which is a new hormonal therapy (9). McNamara et al. reported a transient PSA flare three weeks after the first injection of Ra-223 therapy, and PSA typically decreased at the second injection of Ra-223 (4). PSA and CT did not provide sufficient accuracy in localizing recurrent disease, whereas FDG PET could detect bone marrow metastatic lesions in the present case and is helpful as guidance for biopsy. An increased PSA during Ra-223 therapy may also be explained by contemporary development of bone marrow (in the present case), lymph node, or visceral metastases, in particular with very advanced disease.

Ra-223 was well tolerated with a low risk of myelosuppression. Parker et al. reported grade 3 or 4 thrombocytopenia in 6% and anemia in 13% of cases (3) according to the Common Terminology Criteria for Adverse Events, version 3.0 (10). The present patient had adequate hematologic function before Ra-223 therapy. During and after Ra-223 therapy, he developed hematologic dysfunction, with grade 3 anemia and grade 3 thrombocytopenia, due to progressive bone marrow involvement by cancer. He never received chemotherapy agents that suppress bone marrow.

Tc-99m based whole body bone scanning is sensitive for bony involvement, and it is usually more sensitive than FDG PET for detecting sclerotic/osteoblastic bone metastases. On the other hand, Zhang et al. reported that Tc-99m based bone scintigraphy is less sensitive for purely osteolytic lesions than FDG PET/CT (11). Pacilio et al. reported a good correlation between Tc-99m based bone scan agents and Ra-223 in patients with prostate cancer (1). Tc-99m based bone scan agents may be a predictive marker of Ra-223 accumulation sites because both of them mimic calcium. In the present case, Ra-223 mainly affected osteoblastic lesions and failed to affect osteolytic and bone marrow metastases. A general decrease in Tc-99m HMDP uptake in bone lesions is concordant with a decreased ALP (bone formation marker indicating osteoblastic activity) level during Ra-223 therapy. Nome et al. reported that radiation by Ra-223 induced cell stunning or killing of highly proliferating osteoblasts surrounding skeletal metastases, which cause the ALP level to reduce (12). Previous studies indicate that chemical biomarker (ALP, PSA) and imaging biomarker (Tc-99m based bone scan) were useful for detection of relapse. But chemical biomarker can never detect the site and extent of metastases. Imaging biomarker (Tc-99m based bone scan) is sometimes difficult to rule out osteolytic lesions because of less intense or decreased uptake. FDG-PET/CT is superior to those biomarkers in terms of detection especially osteolytic or bone marrow metastatic sites and the degree or extent of metabolic activity.

One of the benefits of PET/CT is improved biopsy localization information. FDG PET/CT helps to distinguish active lesions (more aggressive osteolytic and/or bone marrow metastases) from dormant lesions (sclerotic bone metastases) after Ra-223 therapy. FDG PET/CT has clinical impact and leads to changes in clinical management.

The present case suggests that PSA elevation continued during Ra-223 therapy due to bone marrow metastases. Ra-223 was effective for osteoblastic lesions, but not for bone marrow metastases. Bone marrow metastases and osteolytic metastases that could not be detected by Tc-99m based bone scintigraphy were detected by FDG PET/CT. Thus, FDG PET/CT has a role in the detection and management of metastatic progressive castration-resistant prostate cancer treated with Ra-223 due to high glucose metabolism.

## Conflicts of interest

No potential conflicts of interest were disclosed.
